# Nivalenol and Deoxynivalenol Affect Rat Intestinal Epithelial Cells: A Concentration Related Study

**DOI:** 10.1371/journal.pone.0052051

**Published:** 2012-12-14

**Authors:** Giuseppe Bianco, Bianca Fontanella, Lorella Severino, Andrea Quaroni, Giuseppina Autore, Stefania Marzocco

**Affiliations:** 1 Department of Pharmaceutical and Biomedical Sciences, School of Pharmacy, University of Salerno, Fisciano, Italy; 2 Telethon Institute of Genetics and Medicine (TIGEM) Naples, Italy; 3 Department of Pathology and Animal Health, Division of Toxicology, School of Veterinary Medicine, University of Naples “Federico II”, Naples, Italy; 4 Department of Biomedical Sciences, Cornell University, Ithaca, New York, United States of America; Indian Institute of Toxicology Reserach, India

## Abstract

The integrity of the gastrointestinal tract represents a crucial first level defence against ingested toxins. Among them, Nivalenol is a trichotecenes mycotoxin frequently found on cereals and processed grains; when it contaminates human food and animal feed it is often associated with another widespread contaminant, Deoxynivalenol. Following their ingestion, intestinal epithelial cells are exposed to concentrations of these trichothecenes high enough to cause mycotoxicosis. In this study we have investigated the effects of Nivalenol and Deoxynivalenol on intestinal cells in an *in vitro* model system utilizing the non-tumorigenic rat intestinal epithelial cell line IEC-6. Both Nivalenol and Deoxynivalenol (5–80 µM) significantly affected IEC-6 viability through a pro-apoptotic process which mainly involved the following steps: (i) Bax induction; (ii) Bcl-2 inhibition, and (iii) caspase-3 activation. Moreover, treatment with Nivalenol produced a significant cell cycle arrest of IEC-6 cells, primarily at the G_0_/G_1_ interphase and in the S phase, with a concomitant reduction in the fraction of cells in G_2_. Interestingly, when administered at lower concentrations (0.1–2.5 µM), both Nivalenol and Deoxynivalenol affected epithelial cell migration (restitution), representing the initial step in gastrointestinal wound healing in the gut. This reduced motility was associated with significant remodelling of the actin cytoskeleton, and changes in expression of connexin-43 and focal adhesion kinase. The concentration range of Nivalenol or Deoxynivalenol we have tested is comparable with the mean estimated daily intake of consumers eating contaminated food. Thus, our results further highlight the risks associated with intake of even low levels of these toxins.

## Introduction

Mycotoxins are food contaminants produced by secondary metabolism of fungi found primarily in cereal grains and derived products. They are not essential to mold growth but sporadically contaminate crops, causing major economic losses every year. Moreover the consumption of food or feed contaminated by mycotoxins is a potential health hazard for both human and animal health [1], [2]. More than 400 different mycotoxins have been isolated and chemically characterized; those of major medical and agricultural concern are aflatoxins, ochratoxins, trichothecenes, zearalenone and fumonisins. Data from the FAO showed that about 25% of food world production is contaminated by at least one mycotoxin [3].

Trichothecenes mycotoxins are chemically related compounds produced by different fungal genera, including *Fusarium, Pennicillium, Mycothecium*, *Trichoderma*, *Trichothecium*, *Stachybotrys*, *Verticimonosporium*, and *Cephalosporium* [4]. A data collection on the occurrence of *Fusarium* toxins in food in the European Union showed a 57% incidence of positive samples for deoxynivalenol (DON) and 16% for nivalenol (NIV), out of several thousands of samples analysed [5]. Due to their toxic properties and their high stability to heat treatment, the presence of these mycotoxins in the food chain is potentially hazardous to health [6]–[9].

The clinical toxicological syndromes caused by ingestion of moderate to high amounts of mycotoxins have been well characterized: they range from acute mortality to slowed growth, and may include reduced reproductive efficiency, gastrointestinal disorders, altered nutritional efficiency [10], [11].

The intestinal epithelial layer represents the first barrier preventing the entry of foreign antigens, including natural toxins, into the underlying tissues. Intestinal epithelial cells (IECs) form a monolayer that constitutes a dynamic and selective barrier which mediates the transport of a variety of molecules. The intestinal epithelium is normally subjected to deformation due to diverse physical forces, including peristalsis during normal gut function. As a consequence of their exposed location, IECs have developed a variety of mechanisms, aside from physical, which act to reduce the toxicity of chemicals and the possible invasion by foreign agents (e.g. protective mucus, antimicrobial peptide secretion, epithelial wound healing and cytokine synthesis) [12]. Long-term NIV chronic exposure in mice induced a reduced body gain and feed efficiency, and an increase in relative organ weight or severe leucopenia [13]. NIV, associated with other trichothecenes, has been shown to correlate with the high incidence of oesophageal cancer in China [14], [15]; those authors also observed papillomas and carcinomas in 47% of mice topically exposed to NIV during 60 weeks. At the cellular level, several reports indicated that DON could alter barrier function [16]–[18], IECs differentiation and the uptake of nutrients [19]. It has also been shown that, as other trichothecenes, NIV and DON are particularly potent inhibitors of protein synthesis and they are able to interfere primarily with the high proliferation rate of cells in tissues such as spleen, bone marrow, thymus and intestinal mucosa. To our knowledge, the effects of NIV on non tumorigenic cells of intestinal origin has been never studied and only few data exist on the effect of NIV and DON, at low doses, on intestinal homeostasis [16], [20], [21]. Thus, the aim of this study was to test the effects of variable concentrations of NIV and/or DON on the non tumorigenic intestinal epithelial cells, IEC-6.

## Materials and Methods

### Reagents

Unless stated otherwise, all reagents and compounds were obtained from Sigma Chemicals Company (Sigma, Milan, Italy).

### Preparation of Trichothecenes Mycotoxins

NIV and DON 10 mM stock solutions of were prepared by dissolution in ethanol and methanol. Then we diluted such solutions with medium obtaining working solutions subsequently divided in several aliquots and stored at –20°C. Before every experiment a fresh aliquot of NIV and DON was used. NIV and DON vehicle was present in cellular medium at concentration lower than 0.5%. The same concentrations of vehicle was added to controls in all experiments.

### Cell Culture

The IEC-6 cell line (CRL-1592) was purchased from the American Type Culture Collection (ATCC, Rockville, MD, USA). IEC-6 cell originated from normal rat intestinal crypt cells [22]. This non-tumorigenic cell line was cultured using Dulbecco’s modified Eagle’s medium (4 g/L glucose) supplemented with 10% (v/v) heat-inactivated fetal bovine serum, 2 mm L-glutamine, 1.5 g/L NaHCO_3_, and 0.1 unit/ml bovine insulin. Cells were used at the 17^th^–21^st^ passage.

### Cell Viability Assay

Cells (5×10^3^) were plated in 96-well microtiter plates and allowed to adhere. Thereafter cells were synchronised for 24 h in serum free media before being exposed to NIV or DON (0.5–80 µM), alone or in combination, for further 24 h in DMEM serum free. Cell viability was then assessed as previously reported using the MTT assay [23], [24]. Briefly, 25 µL of MTT (5 mg/mL) were added and the cells were incubated for 3 h. Thereafter, cells were lysed and the dark blue crystals solubilised with 100 µL of a solution containing 50% (v:v) N,N-dimethylformamide, 20% (w:v) SDS with an adjusted pH of 4.5. The optical density (OD) of each well was measured with a microplate spectrophotometer (Titertek Multiskan MCC/340) equipped with a 620 nm filter. IEC-6 viability in response to treatment with NIV and DON was calculated as: % dead cells = 100-(OD treated/OD control) ×100.

### Analysis of Apoptosis and Cell Cycle Distribution

Hypodiploid DNA was analyzed using PI staining by flow cytometry [25]. Briefly, IEC-6 (3.5×10^5^) cells were grown in 24-well plates and allowed to adhere. Thereafter cells were synchronised for 24 h in serum free media before being exposed to NIV or DON (0.5–80 µM) for further 24 h, in DMEM serum free. Following treatment, culture medium was removed, cells washed once with PBS and then resuspended in 500 µL of a solution containing 0.1% (w/v) sodium citrate, 0.1% Triton X-100 and 50 µg/mL propidium iodide (PI).

Culture medium and PBS were centrifuged and cell pellets were pooled with cell suspension to retain both dead and living cells for analysis. After incubation at 4°C for 30 min in the dark, cell nuclei were analyzed with a Becton Dickinson FACScan flow cytometer using the CellQuest program and the DNA content of the nuclei was registered on a logarithmic scale. Cellular debris was excluded from the analysis by raising the forward scatter threshold, then the percentage of cells in the hypodiploid region (sub G_0_/G_1_) was calculated.

To elucidate apoptosis caspases involvement, in some experiments a broad-spectrum caspase inhibitor, zVAD-fmk (50 µM) was added to IEC-6 cells 30 min before NIV or DON (40 and 20 µM, concentration which gives a medium level of apoptosis). Data are expressed as the percentage of cells in the hypodiploid region.

The effect of NIV or DON on the cell cycle was tested at the concentration of 10 µM [26]. This concentration was chosen because it does not induce a too high level of apoptosis that could result in different number of cells analysed during experiments. IEC-6 cells were seeded in a 12-wells plastic plate at 7.0×10^5^ cells/well and allowed to adhere. Thereafter cells were synchronised for 24 h in serum free media before being exposed to NIV or DON at the concentration of 10 µM for 24 h, in DMEM serum free. After incubation, IEC-6 cells were harvested and fixed in cold 70% ethanol at −20°C. Cell cycle profiles were evaluated by DNA staining with PI (2.5 mg/ml) in phosphate-buffered saline (PBS) supplemented with 100 U/mL ribonuclease A, for 30 min at room temperature. Samples were analysed with a FACScan flow cytometer (Becton Dickinson, CA) using Mod FitLT program.

### Confocal Microscopy: Nuclei Analysis

Cells (3.5×10^5^/well) were plated on 12 mm glass coverslips in 24 well plates and allowed to adhere, thereafter cells were synchronised for 24 h in serum free media before being exposed to NIV or DON (0.5–20 µM) for 24 h, in DMEM serum free. Cells were then fixed in p-formaldehyde (4% v/v in PBS) for 30 min. Cells were permeabilized in Triton X-100 (0.1% v/v in PBS) for 10 min, and then incubated with DAPI at a concentration of 1 µg/mL in PBS for 30 min. The coverslips were mounted with Vectashield (Vector Laboratories, Burlingame, CA, USA). A Zeiss LSM 710 Laser Scanning Microscope (Carl Zeiss MicroImaging GmbH, Jena, Germany) was used for data acquisition. To detect nucleus and filaments, samples were excited with a 458 nm Ar laser. Samples were vertically scanned from the bottom of the coverslip with a total depth of 5 mm and a 63× (1.40 NA) Plan-Apochromat oil immersion objective. A total of 10 z-line scans with a step distance of 0.5 µm were collected and single planes or maximum intensity projections were generated with Zeiss ZEN Confocal Software (Carl Zeiss MicroImaging GmbH).

### Confocal Microscopy: Cytoskeleton Analysis

To study the effects of mycotoxins at non-apoptotic concentrations on cellular cytoskeleton, NIV and DON were tested at concentrations between 2.5 and 0.5 µM. Cells (3.5×10^5^/well) were plated on 12 mm glass coverslips in 24 well plates and allowed to adhere; therefore cells were synchronised for 24 h in serum free media before being exposed to NIV or DON for 24 h, in serum free DMEM. Cells were then fixed in p-formaldehyde (4% v/v in PBS) for 30 min. Cells were permeabilized in Triton X-100 (0.1% v/v in PBS) for 10 min, and then incubated with FITC-conjugated anti-F-actin (Phalloidin-FITC, Sigma) at the concentration of 1µg/mL in PBS for 30 min. The coverslips were mounted with Vectashield (Vector Laboratories, Burlingame, CA, USA). A Zeiss LSM 710 Laser Scanning Microscope (Carl Zeiss MicroImaging GmbH, Jena, Germany) was used for data acquisition. To detect nuclei and filaments, samples were excited with a 488 nm Ar laser. Samples were vertically scanned from the bottom of the coverslip with a total depth of 5 mm and a 63× (1.40 NA) Plan-Apochromat oil immersion objective. A total of 10 z-line scans with a step distance of 0.5 µm were collected and single planes or maximum intensity projections were generated with Zeiss ZEN Confocal Software (Carl Zeiss MicroImaging GmbH).

### Western Blot Analysis

IEC-6 cells (6×10^5^/P60) were plated and allowed to adhere at 37°C in a 5% CO_2_ atmosphere for 24 h. The medium was replaced with serum-deprivated medium and after 24 h NIV or DON (0.5–20 µM) were added and cells incubated for additional 24 h.

Cells were lysed in lysis buffer (50 mM Tris–HCl pH 7.4, with 1% Triton X-100, 150 mM NaCl, 5 mM EDTA, 1 mM phenylmethylsulfonyl fluoride, 2 mM N-ethylmaleimide, 50 mM NaF, 2 mM Na_3_VO_4_, 5 µg/mL aprotinin and 5 µg/mL leupeptin). Protein content was estimated according to Biorad protein assay (BIO-RAD, Milan, Italy) and 50 µg protein/lane were loaded onto an acrylamide gel and separated by SDS-PAGE under denaturating conditions. The separated proteins were then transferred electrophoretically (200 mA per blot 45 min; Trans Blot Semi-Dry, BIO-RAD) to nitrocellulose paper (Immobilon-NC, Millipore, Bedford, USA) soaked in transfer buffer (25 mM Tris, 192 mM glycine, Sigma-Aldrich) and 20% methanol vol/vol (Carlo Erba, Milan, Italy). Non specific binding was blocked by incubation of the blots in 5% no fat dry-milk powder (BIO-RAD) in TBS/0.1% Tween (25 mM Tris; 150 mM NaCl; 0.1% Tween vol/vol, Sigma-Aldrich) for 60 min. After washing, the blots were incubated overnight at 4°C with the primary antibody anti-Bax, anti-Bcl-2, anti-procaspase, anti-caspase-3 for apoptosis studies and anti-connexin-43 (Cx43), anti-phospho-connexin-43, anti-Focal Adhesion Kinase (FAK) and anti-phosho-Focal Adhesion Kinase for cell migration studies; α-tubulin was used as reference protein (all from Santa-Cruz Biotechnology, D.B.A. ITALIA s.r.l, Milan, Italy). After incubation with the primary antibodies and washing in TBS/0.1% Tween, the appropriate secondary antibody, either anti-mouse (diluted 1∶1000) or anti-rabbit (diluted 1∶5000), was added for 1 h at room temperature. Immunoreactive protein bands were detected by chemiluminescence using enhanced chemiluminescence reagents (ECL) and exposed to Hyperfilm (both from Amersham Biosciences, Milan, Italy). Films were then subjected to densitometric analysis using a Gel-Doc 2000 system (BIO-RAD).

### Wound-healing Assay

IEC-6 cells were seeded in a 12-well plastic plate at 1.8×10^5^ cells per well and allowed to adhere; after 24 h of serum deprivation a wound was produced at the centre of the monolayer by gently scraping cells with a sterile plastic p200 pipette tip. Then the incubation medium was removed and cells washed with PBS and incubated with NIV and DON, alone or in combination, at non apoptotic concentrations (0.5–2.5 µM).

The wounded cell cultures were then incubated at 37°C in a humidified and equilibrated (5% v/v CO_2_) incubation chamber of an Integrated Live Cell Workstation Leica AF-6000 LX. A 10× phase contrast objective was used to record cell movements with a frequency of acquisition of 10 min. The migration rate of individual cells was determined by measuring the distances covered from the initial time to the selected time-points (bar of distance tool, Leica ASF software). For each condition three independent experiments were performed. For each wound three different positions were registered, and for each position ten different cells were randomly selected to measure the migration distances. Statistical analyses were performed using GraphPad Prism 4 software (GraphPad, San Diego, CA).

### Data Analysis

All the values shown in tables, figures and text are expressed as mean ± standard error of the mean of at least three independent experiments in triplicate. Data sets were examined by one-way analysis of variance and individual group means were then compared with Bonferroni or Student’s unpaired t-test. A P-value less than 0.05 was considered as significant.

## Results

### NIV and DON Affect IEC-6 Viability

After incubation of IEC-6 cells with graded concentrations of NIV or DON alone or in combination (0.5–80 µM) for 24 h, cell viability was determined using the MTT assay. Control cells viability was designated as 100% and results have been expressed as the concentration of each mycotoxin which induces 50% of mortality in IEC-6 (IC_50_). Both mycotoxins exerted a significant effect on cell viability and had anti-proliferative activity on IEC-6 (IC_50_ value for NIV: 17.75±0.47 µM; IC_50_ value for DON 50.82±0.85 µM, P<0.001vs control). In particular NIV exerted a stronger anti-proliferative activity compared to DON (P<0.001). Moreover, the presence of NIV and DON together in the incubation medium did not induce either additive or synergistic effects on IEC-6 viability (IC_50_ value for NIV+DON: 18.90±0.59 µM).

### NIV- and DON-induced Apoptosis in IEC-6 Cells

**Figure 1:**
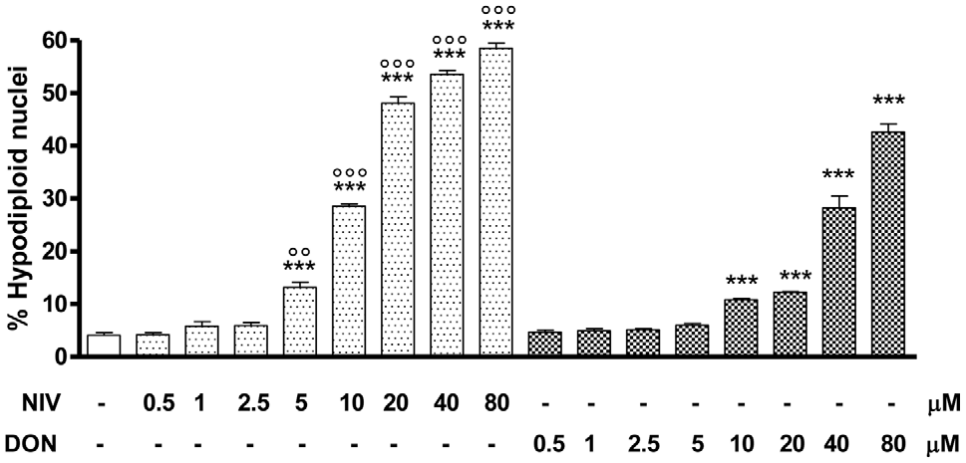
Apoptosis detection by propidium iodide (PI) staining of hypodiploid nuclei after IEC-6 incubation with NIV or DON (0.5–80 µM) for 24 h. Both NIV and DON exibited a significant and concentration-related pro-apoptotic effect on IEC-6 at concentrations higher than 5 µM and 10 µM respectively (***P<0.001 *vs* control). NIV at the concentrations between 5 and 80 µM exibits a stronger pro-apoptotic effect compared to the same concentrations of DON (°°°P<0.001, °°P<0.01 *vs* DON). Data are expressed mean ± s.e.m. from at least three-independent experiments.

In order to investigate the mechanism(s) underlying cell observed decrease in cell viability observed in NIV- or DON- treated IEC-6 cells a cytofluorimetric analysis was performed by incubating IEC-6 with graded concentrations of each mycotoxin (0.5–80 µM) for 24h. Apoptosis was evaluated by cytofluorimetric analysis of PI stained hypodiploid nuclei (Fig. 1). Our results indicate that both NIV and DON significantly (P<0,001) induced apoptosis in IEC-6, in a concentration-dependent manner, from 5 µM and 10 µM for NIV- or DON-treated cells respectively; whereas any apoptotic effect have been observed at the lowest concentrations tested (2.5 µM for NIV and 5 µM for DON). Interestingly the NIV pro-apoptotic effect at concentrations of 10–80 µM was significantly higher than DON’s, indicating a stronger pro-apoptotic activity for NIV. Similarly to the effect observed on cell viability, no additive or synergistic effects on IEC-6 apoptotis were observed after incubation with NIV and DON together (data not shown).

### Induction of Nuclear Morphological Changes in IEC-6 by NIV and DON

**Figure 2:**
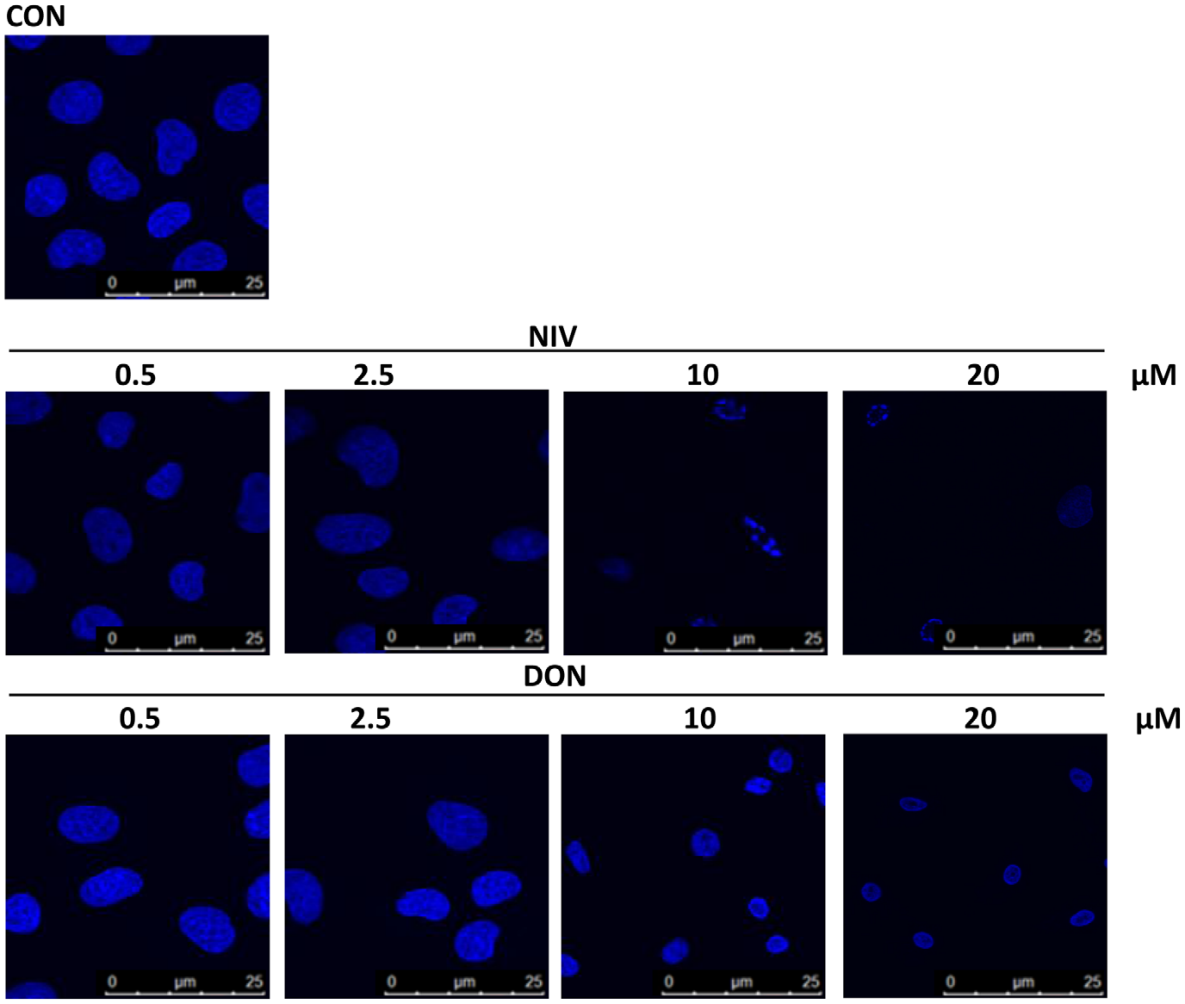
Apoptotic nuclear morphological changes highlighted by DAPI staining in cells treated with graded concentration of NIV and DON (0.5–20 µM) for 24 h. Apoptotic cells showed pyknotic nuclei and apoptotic bodies were clearly identified after incubation with higher concentration of NIV while DON induces a less marked effect. Cultures were examined and photographed using a confocal microscope as described in method section.

In order to confirm induction of apoptosis and perturbation in cell morphology due to the trichothecenes NIV or DON, IEC-6 nuclei were also observed after DAPI staining (Fig. 2). At the lowest concentrations tested (0.5 and 2.5 µM) cell nuclei appeared similar to untreated cells. Apoptotic cells were clearly identified by the appearance of pyknotic nuclei and apoptotic bodies (condensed and fragmented nuclei) after incubation with the highest NIV concentrations tested (10–20 µM). The same DON concentrations (10–20 µM) induced a lesser effect. For these experiments, to better represent the results obtained, NIV and DON were tested at concentrations not so far from NIV IC_50_.

### Induction of Cell Cycle Arrest by NIV and DON

**Figure 3:**
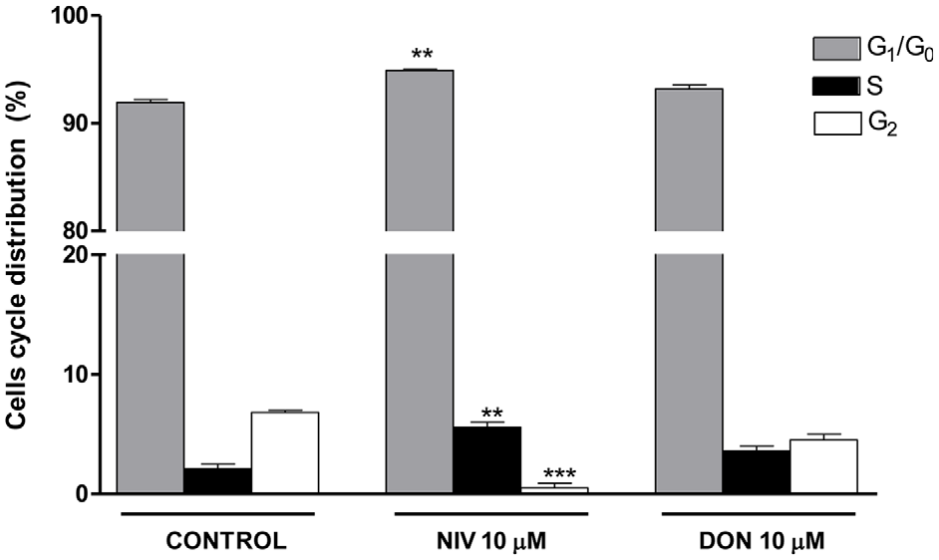
Flow cytometric analysis of IEC-6 cycle phase distribution. Cells were treated with either NIV or DON (10 µM) for 24 h, incubated with PI and analysed for cell cycle analysis using a Becton Dickinson FACScan flow cytometer and ModFit software (***P<0.001,**P<0.01 *vs* control). Data are expressed mean ± s.e.m. from at least three-independent experiments.

In order to determine if NIV and DON could also affect cell cycle distribution we carried out a cell cycle analysis using a mycotoxin concentration which does not induce a strong apoptotic effect. We treated IEC-6 with 10 µM of each mycotoxin for 24 h. As showed in Fig. 3, exposure for 24 h to NIV (10 µM) resulted in a significant increase of the G_0_/G_1_ and S phase cell cycle distribution (P<0.01 vs control) accompanied by a significant (P<0.001 vs control) decrease in G_2_ phase compared to untreated cells. No significant changes in cell cycle profile was observed in DON-treated cells.

### NIV and DON Affects Bax and Bcl-2 Expression IEC-6

**Figure 4:**
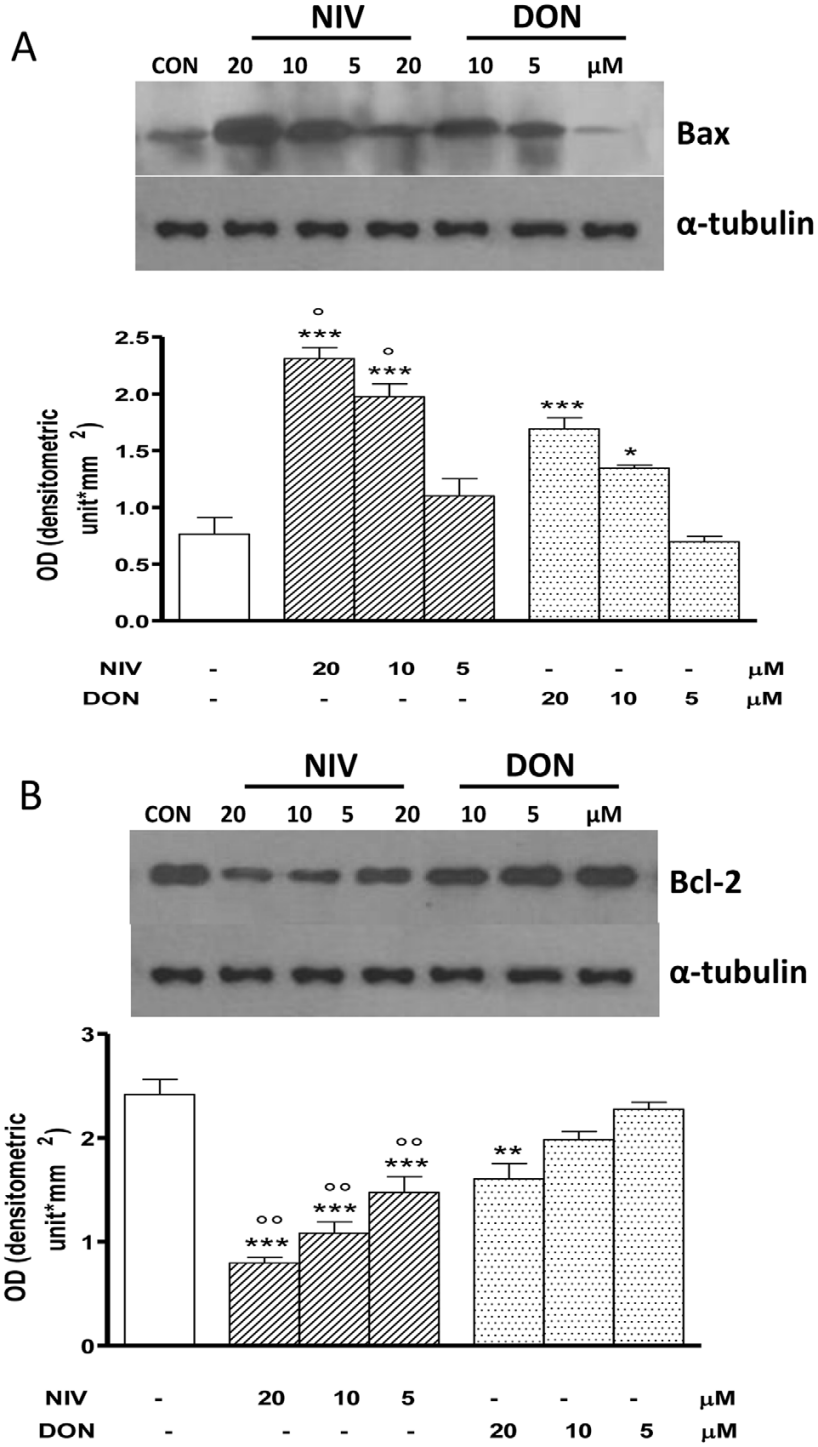
Concentration dependent effect in NIV and DON treated cells on apoptosis-related proteins Bax (panel A) and Bcl-2 (panel B) expression. Cells were treated with increasing concentration of NIV or DON (5–20 µM) for 24 h and protein expression was detected by Western blot. Tubulin protein expression was used as a loading control. (***P<0.001,**P<0.01,*P<0.05 *vs* control; °°P<0.01,°P<0.05 vs DON). Western blot is representative of at least three-independent experiments.

The pro-apoptotic protein Bax and the anti-apoptotic protein Bcl-2 are involved in apoptotic process. In order to examine the effect of NIV or DON on these proteins their expression was assayed in IEC-6 exposed to tricothecenes by Western blot analysis. Both tricothechenes induced an altered expression of Bax (Fig. 4, panel A) and Bcl-2 (Fig. 4, panel B) compared with the control cells. Bax expression was significantly increased in NIV and DON treated cells at the concentrations of 20 and 10 µM (P<0.05). Bcl-2 expression was significantly decreased in NIV treated cells at all tested concentrations (P<0.001) whereas DON treatment induced a significant increase only at the highest tested concentration (20 µM; P<0.01). Both in Bax increase and in Bcl-2 decrease NIV resulted stronger compared to DON (p<0.05).

### Caspase Activation in NIV and DON Treated IEC-6

**Figure 5:**
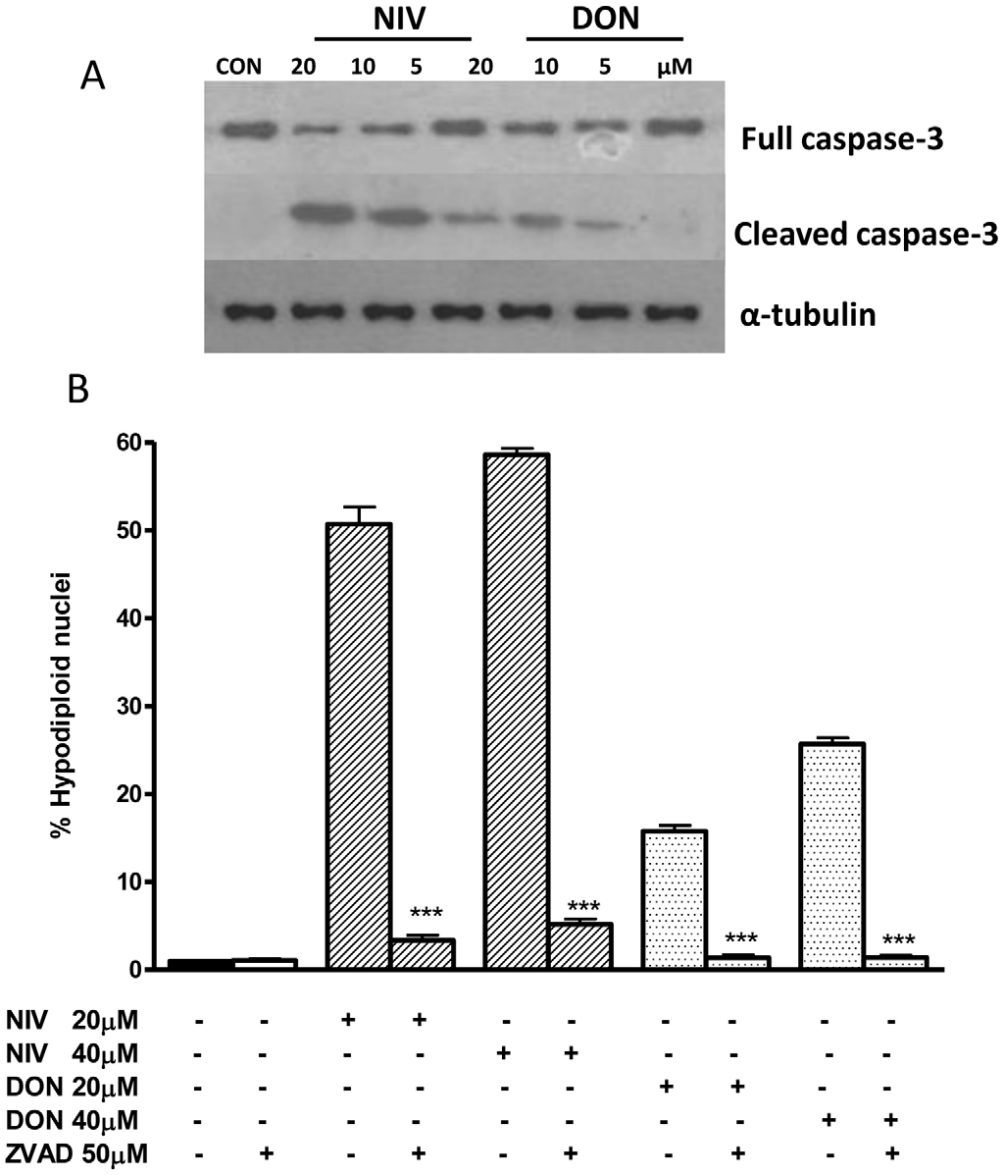
Concentration dependent effect in NIV and DON treated cells on caspase-3 (full and cleaved form) expression. Cells were treated with increasing concentration of NIV or DON (5–20 µM) for 24 h and protein expression was detected by Western blot. Tubulin protein expression was used as a loading control. The Western blot is representative of independent experiments (panel A). Apoptosis detection by propidium iodide (PI) staining of hypodiploid nuclei with a broad spectrum caspase inhibitor (zVAD-fmk) (panel B). IEC-6 were treated with NIV or DON (20 and 40 µM) for 24 h in presence of zVAD-fmk (50 µM). The pro-apoptotic effect of NIV or DON was significantly reduced by the caspase inhibitor (***P<0.001 NIV+zVAD *vs* NIV alone; ***P<0.001 DON+zVAD *vs* DON alone). Data are expressed mean ± s.e.m. from at least three-independent experiments.

Caspase-3 activation by proteolytic cleavage is a marker and an irreversible step in the apoptosis cascade [26]. Treatment with DON, and even more with NIV-treated IEC-6 led to a marked increase in caspase-3 expression, associated with a decrease in pro-caspase-3, the inactive form of caspase-3 (Fig. 5, panel A). In order to determine whether caspase activation could contribute to apoptosis induced in IEC-6 by NIV and DON, we exposed cells to trichothecenes in presence of zVAD-fmk, a broad-spectrum inhibitor of caspases. As shown in Fig. 5, panel B, zVAD-fmk (50 µM), significantly (P<0.001) reduced the percentage of hypodiploid nuclei, indicating a role of caspase activation in NIV and DON induced apoptosis.

### NIV and DON Reduce IEC-6 Migration

**Figure 6:**
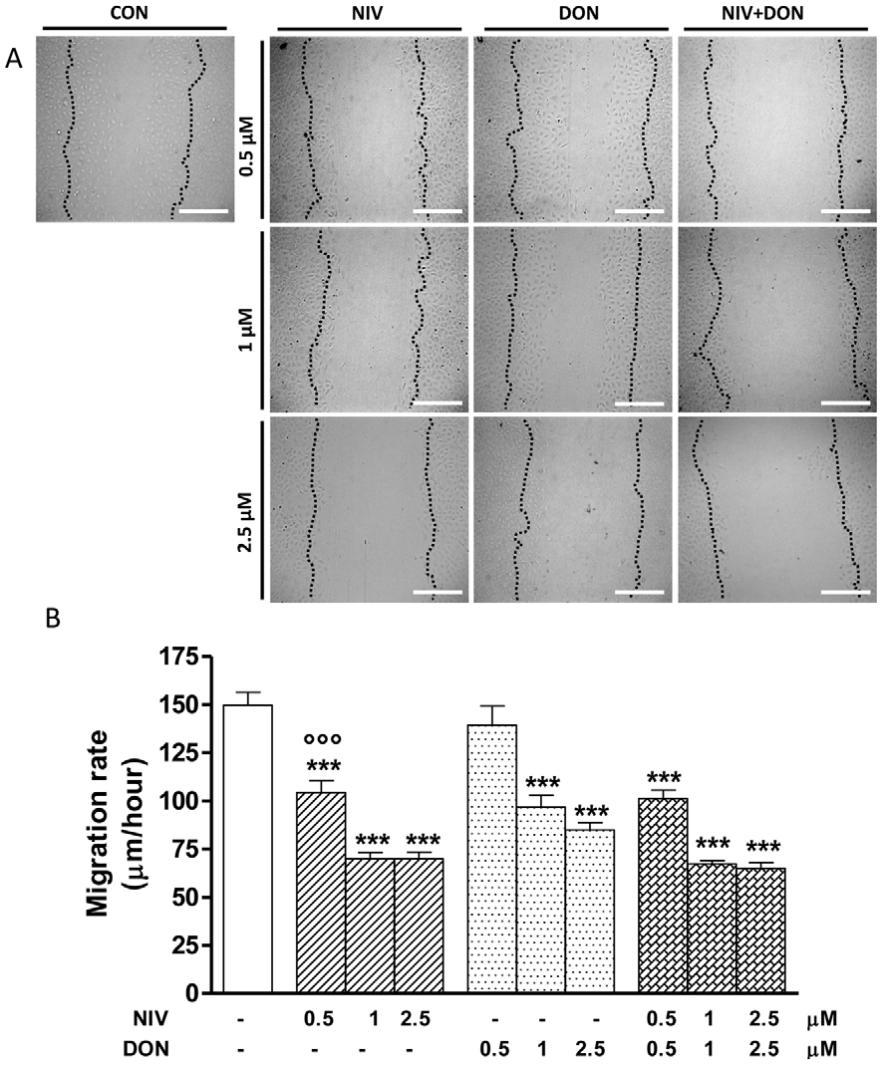
Representative pictures of wound repair from mechanical scratch 24 h after NIV and DON cells treatment (0.5–2.5 µM). Black dotted-line indicates the edge of the wounded area at the starting time (panel A). Quantitative analysis of the wound repair 24 h after making the scratch. For each condition ten different cells were randomly selected to measure the migration distances covered every 10 min from the initial time up to the end of incubation time (***P<0.001 *vs* control; °°°P<0.001 vs DON; panel B). Data are expressed mean ± s.e.m. from at least three-independent experiments. Bar  =  200 µM.

To determine if NIV or DON at non-apoptotic concentrations influence intestinal epithelial cells homeostasis, we performed a wound-healing assay on IEC-6 monolayers in the presence of graded concentrations (0.5–2.5 µM) of each mycotoxin alone or in combination. The confluent cultures were scraped to create a wound and cell migration was monitored by time-lapse video-microscopy at the site of the wound for 24 h. We measured the migration distances of selected cells at different time points. Figure 6 shows a progressive concentration-related slowing down in migration speed of cells treated with NIV or DON compared to untreated cells. Also in these experiments NIV was more powerful than DON (Fig. 6, panel B) in reducing IEC-6 restitution leading to a stronger reduction of cell migration at the lowest tested concentration. No additive or synergic effects on wound repair was observed in restitution after IEC-6 treatment with NIV and DON together.

### Trichothecenes Induce IEC-6 Actin Cytoskeleton Rearrangement

**Figure 7:**
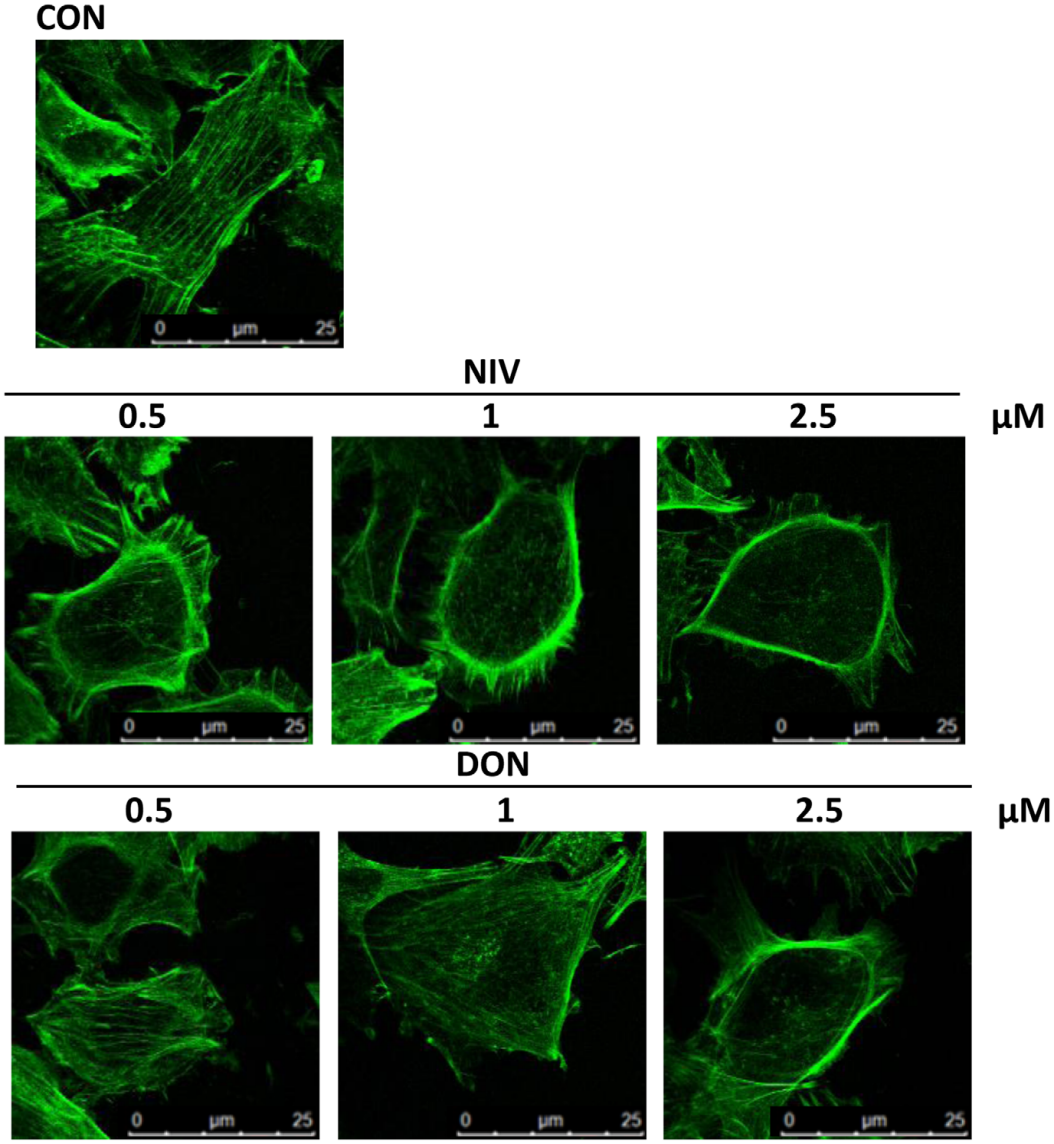
Effect of mycotoxins NIV and DON on IEC-6 cytoskeleton highlighted by FITC-coniugated phalloidin staining. The microtubule cytoskeleton is displayed in control IEC-6. The microtubules are evident as actin filaments that originate near the nucleus and radiate through the cytosol. Incubation for 24 h of cultured cells with NIV and DON (0.5–2.5 µM) caused a reorganisation of actin filaments characterised by redistribution to the cell subcortical compartment and subsequent cell rounding in concentration dependent manner. Cultures were examined and photographed using a confocal microscope as described in method section.

As actin stress fibers play a crucial role in the remodelling, stretching and migration of epithelial cells, we examined the effect of NIV or DON on the assembly of actin stress fibers in the cells at the wound front, in order to investigate the mechanisms of trichothecenes inhibition on IEC-6 epithelial wound repair. After epithelial wound generation, the F-actin microfilaments were labeled with fluorescein-conjugated phalloidin, as described in the Method section. Untreated cells showed an intact actin cytoskeleton with a thick actin cortex and thin visible stress fibers traversing the cytosol; actin stress fibers appeared diffusely present in the cytoplasm of the migrating cells at the wound front, also characterized by stretching and elongation (Fig. 7). In contrast, the addition of DON and, more markedly of NIV, caused a reorganisation of actin filaments characterised by their redistribution to the cell subcortical compartment and subsequent cell rounding in a concentration dependent manner (Fig. 7).

### NIV and DON Induce Changes in Gap Junction, Connexin-43 and Focal Adhesion Kinase Expression

**Figure 8:**
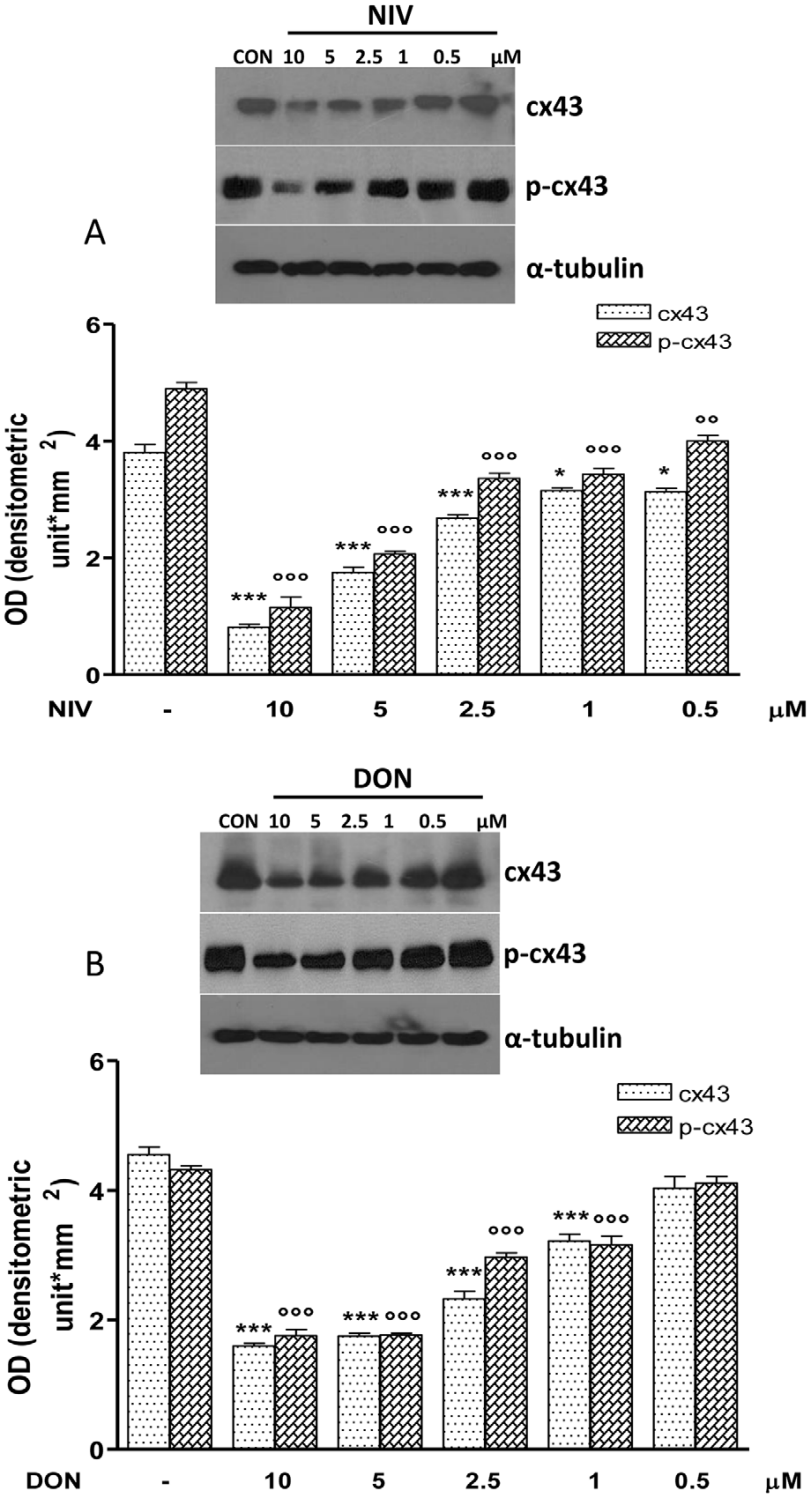
Concentration dependent effect in NIV (Panel A) and DON (Panel B) treated cells on Cx43and its phosphorilated form (pCx43) expression. Cells were treated with increasing concentration of NIV or DON (0.5–10 µM) for 24 h and protein expression was detected by Western blot (***P<0.001,*P<0.05 *vs* control; °°°P<0.001,°°P<0.05 *vs* DON). Tubulin protein expression was used as a loading control. The Western blot is representative of at least three-independent experiments.

**Figure 9:**
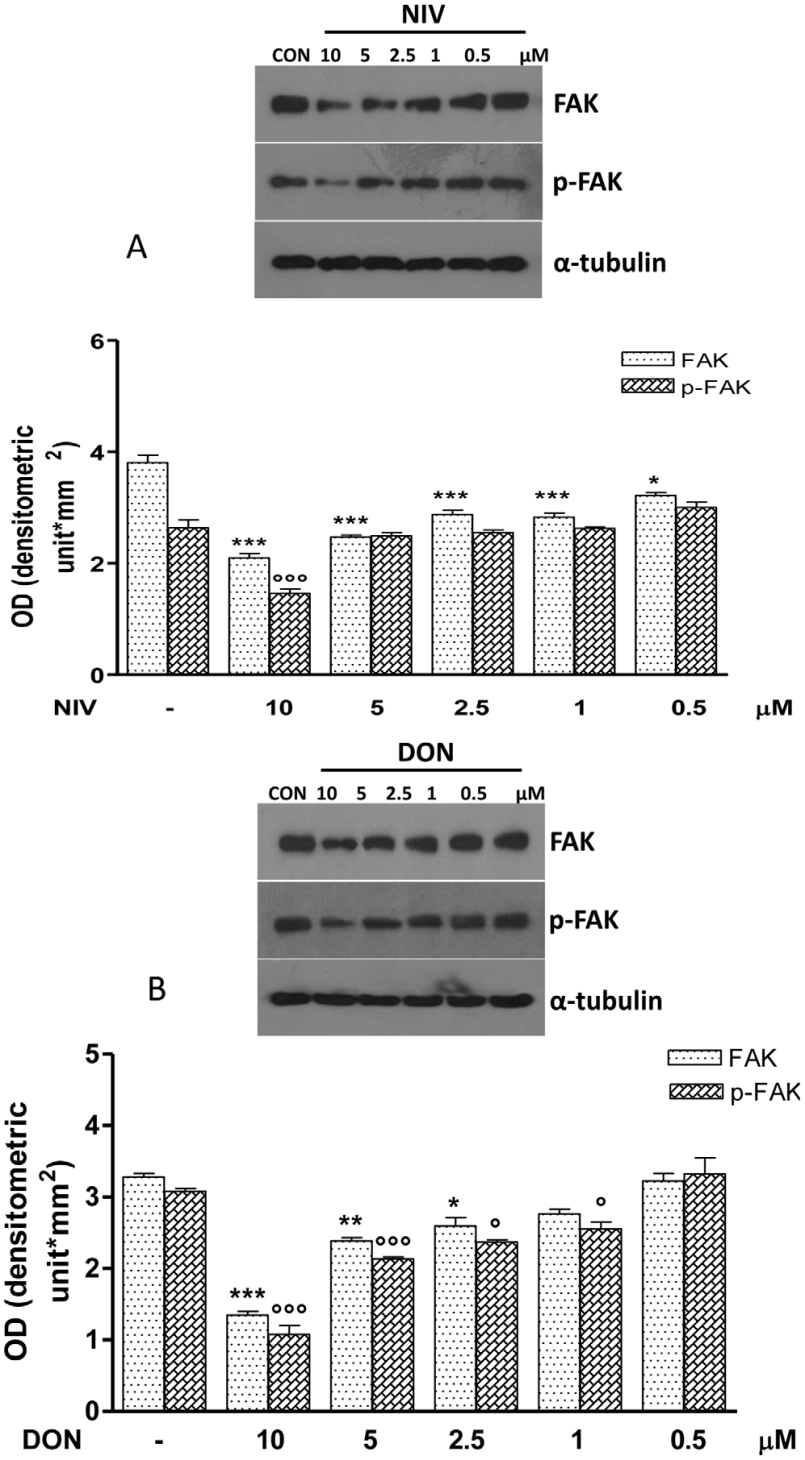
Concentration dependent effect in NIV (Panel A) and DON (Panel B) treated cells on FAK and its phosphorilated form expression (pFAK). Cells were treated with increasing concentration of NIV or DON (0.5–10 µM) for 24 h and protein expression was detected by Western blot. (***P<0.001, **P<0.01, *P<0.05 *vs* control; °°°P<0.001, °P<0.05 vs DON). Tubulin protein expression was used as a loading control. The Western blot is representative of at least three-independent experiments.

Because enterocytes migrate together, mucosal healing may require inter-enterocyte communication via gap junctions. Western blotting analysis showed the Cx43 (Fig.8) and FAK (Fig. 9) contribute in the reduced migration observed in NIV or DON treated IEC-6 cells. Cx43 and pCx43 were significantly reduced in IEC-6 cells treated with NIV at all assayed concentrations (10–0.5 µM P<0.05) while DON treatment reduces Cx43 and pCx43 at concentrations between 10 and 1 µM (P<0.001; Fig.8). Similar effects were observed on FAK and pFAK expression: both NIV and DON significantly reduced FAK and pFAK expression but, as for Cx43, this effect was stronger for NIV compared with DON (Fig. 9).

## Discussion

The importance of studying the trichothecenes NIV and DON is related to food and feed contamination, which induces severe consequences on both human and animal health.

The gut mucosa is the first and the principal site of mycotoxins exposure and toxic action and constitutes the first barrier after their ingestion. Human microflora seems to be unable to produce de-epoxidated metabolites of NIV and a close relationship between the dietary exposure and DON recovery in human and animal urine samples has been reported [28], [29]. Studies performed on human enterocytes confirmed that trichothecenes, such as DON, easily cross the intestinal monolayer through passive or facilitated diffusion, with a possible contribution of paracellular passage [17], [30]. Therefore, IECs could contribute to NIV and DON toxicity by allowing their entry into the systemic circulation and thus their transport to the whole body.

While little was previously known about the effects of NIV in cultured intestinal epithelial cells, in this study we demonstrated that NIV and DON affect IEC-6 cells homeostasis at concentrations plausibly encountered in the gastrointestinal tract after consumption of contaminated food, with significant effects even at concentrations 10 fold lower, acting through different mechanisms. Our data indicate that DON, and more so NIV, exert a significant effect on IEC-6 cell viability. Food and feed commodities are often contaminated by more than one mycotoxin [31]; among the several combinations that frequently occur, NIV and DON are often mentioned [32]. In our study we report that the contemporary presence of NIV and DON in the culture medium added to IEC-6 cells didn’ t lead to a synergic effect on cell viability thus indicating that their co-presence didn’t enhance each own individual effect, in accordance with previous studies on different cell systems [33], [34]. In order to investigate if the reduced IEC-6 viability due to treatment with NIV and DON could be related to apoptosis induction we examined hypodiploid nuclei by cytofluorimetric analysis after PI staining. The results of our study indicate that NIV and DON induce apoptosis in IEC-6 by mechanisms involving: (i) cellular morphological changes, (ii) hypodiploid nuclei forming, (iii) the induction of the pro-apoptotic protein Bax; (iv) the inhibition of the anti-apoptotic protein Bcl-2 and (v) the induction of caspase-3 activation. Cell cycle analysis gave another insight into the mechanism of the effects of these mycotoxins on the IEC-6 proliferation. In particular we have also observed that NIV treatment induces in IEC-6 a significant arrest of cell cycle in G_0_/G_1_ and G_2_; there was also an increase in S phase. On the contrary no differences in cell cycle phase distribution were observed after 24 h DON treatment, as reported in IPEC-J2 [35].

Our data on NIV and DON ability to induce apoptosis are also in accordance with previous studies, which demonstrated the pro-apoptotic effect of these trichothecenes both in intestinal and not-intestinal cell lines [33], [34]. Interestingly our results indicated that NIV-induced apoptosis was greater than for DON at the same concentration range, as we previously reported in cultured macrophages [34]. Moreover, as for cell viability, no additive or synergistic effects on IEC-6 apoptosis were observed after incubation with NIV and DON together, also in accordance with our previous study on macrophages [34]. Members of the Bcl-2 family, as Bcl-2 and Bax, and are involved in signaling pathways regulating caspase-3 activity necessary for chromatin condensation and DNA fragmentation that characterize apoptosis. Our results showed that DON but greater NIV reduced the anti apoptotic protein Bcl-2 expression and increase the pro-apoptotic protein Bax, respect to control cells. The pro-apoptotic complex, involving Bax, induces pores in the mithocondrial membrane leading to cythocrome C release and caspase-9 activation which activates caspase-3. Caspase-3 is an “executioner” that degrades a variety of cellular components producing apoptosis [34]. In order to evaluate caspase-3 involvement in NIV- and DON-induced apoptosis, we exposed IEC-6 to trichothecenes in presence of zVAD-fmk, a broad-spectrum inhibitor of caspases. Incubation of IEC-6 with zVAD-fmk reverted NIV and DON induced apoptosis indicating an involvement of caspase in this process. Moreover Western blot analysis showed an activation of caspase-3 in macrophages exposed to NIV and DON. The intestinal epithelium when injured by various stimuli (e.g. normal digestion, toxic substances, inflammation, oxidative stress) and undergoes a wound healing process. Intestinal wound healing is dependent on a precise balance between migration, proliferation and differentiation of the IECs adiacent to the wound area [36].

Most IEC-6 migrate during the phase of restitution in the wounded area within 24 hours [37]. Here we report that NIV and DON at not pro-apoptotic concentrations (<5 µM), significantly reduced the restitution process; no additive or synergic effects were observed in the contemporary presence of both mycotoxins on IEC-6 restitution. IECs are physically and functionally interconnected via membrane channels that are composed of the gap junction protein Cx43 regulating their migration [37]. Gap junctions exist between adjacent cells and they allow the transfer of small molecules (under 1000 daltons) between adjoining cells. Each gap junction channel is comprised of a pair of hexameric arrays of individual subunits called connexins, of which the most widely expressed isoform is connexin-43 (Cx43) [38], [39]. The function of gap junctions is regulated in part through phosphorylation of individual connexin molecules, which serves to regulate the localization of the channels at the plasma membrane as well as to regulate the channel through gating [40], [41]. Our evidence indicates that the exposure of IEC-6 to NIV or DON leads to a marked reduction in gap junction communication, reducing both Cx43 and its phosphorylated isoform (pCx43), leading to an impaired epithelial migration and thus to an impaired barrier repair. Our data are confirmed by a another study which demonstrated the ability of DON to increase the paracellular permeability of Caco-2 cell monolayers and to reduce the expression of tight junction constituent protein claudin-4 and protein synthesis [42].

Various factors have been shown to stimulate epithelial restitution and proliferation and, among these, activation of focal adhesion kinase (FAK) has been linked to gastric wound healing. FAK is a non-receptor tyrosine kinase involved in adhesion signalling in multiple cell types, including those of epithelial derivation. FAK has an established role in many cellular processes involved in intestinal homeostasis, including cell proliferation, migration and survival [43]. Through its kinase activity, FAK provides robust anti-apoptotic signals [44]. Expression of dominant-negative FAK mutants in intestinal epithelial cell lines leads to an increased apoptosis due to the loss of adhesion-mediated survival signals [45], [46]; conversely, FAK over-expression has been shown to suppress apoptosis [47]. Our data show that DON and even more strongly NIV significantly reduced FAK expression in IEC-6. The IEC-6 cells were originally derived from the rat proximal small intestine. When confluent, these cells form monolayers that morphologically resemble normal small intestinal cells and exhibit a highly organized actin cytoskeleton [48], [49]. During IECs restitution extensive reorganisation of the actin cytoskeleton is necessary. DON and more markedly NIV produced a marked decrease in actin stress fibers and a corresponding increase in the density of the cortical actin thus preventing IEC-6 migration.

The amount of NIV in cereal products varies considerably among world regions (from 20–60 µg/kg in France, to 584–1780 µg/kg in China) [15], depending on weather and culture conditions [50]. Estimated NIV intake of the French population is 88 ng/kg per day in adults, 163 ng/kg per day in children, and reaches 210 ng/kg per day in vegetarians [51]. In European countries, the daily intake remains below the provisional maximum tolerable daily intake (PMTDI) value of 0.7 µg/kg per day, but in Asia, particularly in Japan and China, the daily intake may exceed the PMTDI. The concentration that we used in this study for NIV is 5 µM (corresponding to about to 1560 µg/Kg) and is consistent with the levels plausibly encountered in the gastrointestinal tract after consumption of heavily contaminated food. This may occur particularly in the case of unfavorable weather conditions or in eastern regions of Asia [15], [52], [53], where particularly *Fusarium* species (e.g. *F. poae and F. crookwellense*) seems to be responsible for heavy contamination of cereals by NIV.

The range of the assayed DON concentrations is also in accordance with the levels plausibly encountered in the gastrointestinal tract after consumption of DON contaminated food. These values have been derived from estimated daily intakes of the SCOOP program [54]. The lower DON concentration (0.16 µg/ml corresponding to 0.5 µM used in this study) corresponds to the mean estimated daily intake of French adult consumers eating food contaminated by mean DON levels, on a chronic basis. The DON concentration of 2 µg/ml corresponds to a high level that can be reached after consumption of heavily contaminated food that can be encountered occasionally (e.g. unfavourable weather conditions) or among children and vegans/macrobiotics [55], [51]. Bony et al. [20] previously reported also a genotoxic potential associated to low levels of NIV and the enhancement of intestinal inflammation by low doses of DON has also been reported [21]. Our data demonstrate that NIV and DON exert a pro-apoptotic effect on IEC-6 but, at low doses, also affect integrity and functioning of intestinal epithelial cells providing evidence for an additional toxic mechanism. While the contemporary presence of NIV and DON has not been associated with a greater toxicity, our results highlighted the stronger toxic effect of NIV respect to DON on IEC. Thus, although less present in contaminating food and feed, our results point out the importance of the toxic effect of NIV further highlighting the risk assessment process of these toxins that are of growing concern.
